# Erratum: Relationship between khat chewing and upper digestive tract cancers among male patients in Hargeisa: a preliminary case-control study

**DOI:** 10.3332/ecancer.2026.2094

**Published:** 2026-03-17

**Authors:** Abdiwahab M Ali, Mirriam N Mutuku, Abdiwahab Hashi, Omar M Muhumed

**Affiliations:** 1 Department of Public Health, School of Postgraduate Studies and Research, Amoud University, Sha'abka, Hargeisa 63252, Somaliland; 2 School of Postgraduate Studies and Research, Amoud University, Sha'abka, Hargeisa 63252, Somaliland

**Keywords:** khat chewing, UDT cancers, risk factors, Somaliland

## Abstract

**Background:**

Khat chewing is a common cultural practice in countries bordering the Red Sea and the east coast of Africa. Despite some indications in the literature, its association with upper digestive tract (UDT) cancers is under-researched.

**Methods:**

This case-control study investigated the relationship between khat chewing and UDT cancers among male patients in Hargeisa, Somaliland. A total of 97 participants (36 cases with UDT cancer and 61 controls) were recruited using purposive sampling from the two cancer clinics in Somaliland: Nageeye Cancer Clinic and Needle Hospital. Data were collected via structured interviewer-administered questionnaires. Analysis was performed using Stata/MP version 16.

**Results:**

After adjusting for confounders, khat chewing was significantly associated with increased UDT cancer risk in a dose-response manner. Ever chewing khat was associated with an adjusted odds ratio (aOR) of 3.51 (95% confidence interval (CI) 1.23–11.2; p = 0.024). Chewing for >20 years had an aOR of 36.8 (95% CI 7.16–303; p < 0.001), daily chewing an aOR of 22.6 (95% CI 5.30–124; p < 0.001) and consuming >600 g per session an aOR of 6.87 (95% CI 2.06–25.8; p = 0.002).

**Conclusion:**

These findings highlight a strong association between khat chewing and UDT cancers and underscore the urgent need for public health interventions, including awareness campaigns and policy measures, to mitigate khat-related cancer risks in Somaliland.

## Background

Khat (*Catha edulis*), is a stimulant plant native to regions along the Red Sea and East Africa, has been traditionally chewed for centuries in various cultures under different names such as chat, qat and miraa [1]. Despite its widespread use primarily in Arabic, Middle Eastern and African communities, the potential health risks associated with khat consumption have gathered increasing attention, particularly concerning its role in upper digestive tract (UDT) cancers [2].

UDT cancers include malignancies affecting organs like the oral cavity, esophagus and stomach, significant contributors to global cancer incidence and mortality, especially prevalent in low- and middle-income countries [3]. Esophageal cancer, in particular, ranks prominently among these cancers, with Eastern Africa identified as a hotspot region for its high prevalence and mortality rates [4]. Despite this geographic variance, the specific mechanisms linking khat consumption to these cancers remain under-researched.

Research indicates that both the duration and frequency of khat chewing play significant roles in cancer development. For instance, studies in Saudi Arabia and Yemen have associated long-term khat chewing with oral squamous cell carcinoma and esophageal cancer, respectively [5, 6]. Additionally, a study in Djibouti found a significant association between khat consumption and premalignant oral lesions [7]. Moreover, the amount of khat consumed in each session has been linked to increased risks of dental health problems and potentially higher susceptibility to UDT cancers [8]. A study by Lukandu *et al* [9] pointed out that there was an association between khat use and oral lesions such as hyperkeratosis and oral cancer. A study conducted in Ethiopia by Dessalegn *et al* [10] concluded that tobacco smoking and khat chewing were positive predictors of esophageal cancer. These findings point out the importance of understanding the health risks associated with khat chewing, particularly in contexts where it is a common cultural practice.

This study aims to address this gap by looking into the relationship between khat chewing and the development of UDT cancers, focusing specifically on oral cavity and esophageal cancers. The objectives include evaluating the duration, frequency and amount of khat chewed as potential risk factors for these cancers among male patients in Hargeisa, Somaliland.

## Methods

This study employed a case-control design to investigate the association between khat chewing and UDT cancers among male patients in Hargeisa, Somaliland. Cases were defined as individuals diagnosed with UDT cancers (oral or esophageal), while controls were selected from the same facilities without such diagnoses. The study utilised non-probability purposive sampling to recruit participants from the two primary cancer clinics in Somaliland: Nageeye Cancer Clinic and Needle Hospital. A total of 97 participants were included in the final analysis, comprising 36 cases and 61 controls.

Data were collected using a structured questionnaire administered via face-to-face interviews, capturing socio-demographic information (age, education, marital status), detailed khat chewing history (duration, frequency and amount) and other potential risk factors such as smoking habits and hot beverage consumption. Medical records were reviewed to validate diagnostic information for cases.

All statistical analyses were performed using Stata/MP version 16.0. Descriptive statistics summarised the socio-demographic and behavioural characteristics, with frequencies and percentages for categorical variables. Differences between cases and controls were assessed using Pearson’s Chi-squared test or Fisher’s exact test for small cell counts (expected frequency < 5).

To evaluate the association between khat chewing and UDT cancers, binary logistic regression models were constructed. Prior to analysis, categories with zero case counts were merged for model convergence: duration categories ‘<5 years’ and ‘5–10 years’ into ‘<10 years,’ and frequency categories ‘Once a week’ and ‘Twice a week’ into ‘≤2 days/week.’ Crude odds ratios (ORs) were calculated via univariate logistic regression and adjusted odds ratios (aORs) via multivariate models, fully adjusted for confounders including age group, education level, marital status, smoking history and hot beverage consumption. A separate analysis assessed the joint effect of khat chewing and smoking compared to neither habit. Results are presented as ORs with 95% confidence intervals (CI), with *p* < 0.05 considered statistically significant.

## Results

A total of 97 male participants were included in the study: 36 cases diagnosed with UDT cancers (oral cavity or esophageal) and 61 controls without such diagnoses, recruited from Nageeye Cancer Clinic and Needle Hospital in Hargeisa, Somaliland.

In adjusted multivariate logistic regression models, ever chewing khat was associated with an increased risk of UDT cancers (aOR 3.51, 95% CI 1.23–11.2; *p* = 0.024). A clear dose-response relationship was observed with duration, frequency and amount of khat chewed.

Chewing khat for more than 20 years was strongly associated with UDT cancer risk (aOR 36.8, 95% CI 7.16–303; *p* < 0.001). Daily chewing carried an aOR of 22.6 (95% CI 5.30–124; *p* < 0.001), while consuming more than 600 g per session was associated with an aOR of 6.87 (95% CI 2.06–25.8; *p* = 0.002) (Table 1, Figure 1).

The majority of participants were aged over 64 years, had no formal education and were married (Table 2). Cases had significantly lower education levels (*p* = 0.015) compared to controls. A significantly higher proportion of cases reported ever chewing khat (83.3% versus 50.8%; *p* = 0.001), chewing for >20 years (55.6% versus 3.3%; *p* < 0.001), daily chewing (58.3% versus 6.6%; *p* < 0.001) and consuming >600 g per session (63.9% versus 16.4%; *p* < 0.001). No significant differences were observed in smoking status, smoking duration, frequency or amount, nor in hot beverage consumption.

An additional model examining the combined effect of khat chewing and smoking showed that individuals who only chewed khat had the highest risk (aOR 6.12, 95% CI 1.51–32.3; *p* = 0.017), followed by those who both chewed khat and smoked (aOR 4.69, 95% CI 1.17–24.4; *p* = 0.041). Smoking alone was not significantly associated with increased risk (aOR 2.86, 95% CI 0.41–20.8; *p* = 0.28) (Table 3).

## Discussion

The findings of this study indicate a significant association between khat chewing and the risk of developing UDT cancers among male patients in Hargeisa, Somaliland. The study revealed that individuals who had ever chewed khat had 3.51 times higher odds of developing UDT cancers compared to non-chewers. This is supported by a systematic review by Chong *et al* [2], which found that khat chewing is associated with an increased risk of oral cancer, particularly in long-term users. Additionally, a pilot case-control study in Ethiopia by Leon *et al* [11] found a strong association between qat (khat) use and esophageal cancer, with odds ratios ranging from 6.4 to 39.9 depending on frequency and duration.

Our study demonstrated a dose-response relationship, where longer duration, higher frequency and larger amounts of khat chewing were associated with progressively higher risks of UDT cancers. Specifically, chewing khat for more than 20 years was associated with 36.8-fold increased odds, daily chewing with 22.6-fold and consumption of >600 g per session with 6.87-fold increased odds. These findings align with a study in Yemen by Al-Abed et al [5], which reported that daily khat chewers had a 5.5 times higher risk of esophageal cancer. Similarly, Al-Jamaei *et al* [12] reported that habitual khat chewing induces premalignant oral lesions in dose- and time-dependent manner. Another study done in Yemen by Al-Maweri *et al* [13] supported the association between khat and oral malignant, as well as potentially malignant oral disorders, highlighting that habitual khat chewing can induce oral erythroplakia, a premalignant lesion.

Moreover, our study’s findings on the dose-response relationship between khat consumption levels and UDT cancers are consistent with a study in Ethiopia by Walle *et al* [8], which found that chewers who consumed 100 g or more of khat in a single session had a 4.33-fold increased risk of oral premalignant lesions compared to those who chewed less. This is supported by another cross-sectional clinical sampling study by Kassie *et al* [14] (*n* = 109) and reported consumption of 100 g of khat per day significantly (*p* < 0.05) increased micronuclei and genetic damage in oral mucosa cells, indicating a dose-dependent rise in genotoxic effects and oral cancer risk among users.

The general study findings are consistent with a study which was done by Al-Maweri *et al* [13] in Yemen, which reported that among 547 khat users, the presence of premalignant oral lesion was significantly associated (*p* < 0.001) with khat chewing only (without smoking). Also agrees with retrospective study by Soufi *et al* [15] (*n* = 28) reported that 36% of the non-smoking, oro-pharyngeal cancer patients had a history of khat chewing for at least 25 years. This study disagrees with a case-control study by Machoki *et al* [16] involving 91 cases and 182 controls, which found no significant association between khat usage and esophageal cancer (p>0.05).

The joint effect analysis revealed that khat chewing alone was associated with a 6.12-fold increased risk, while combined khat chewing and smoking had a 4.69-fold risk, and smoking alone was not significant. This suggests that in this population, khat chewing may be the primary driver of UDT cancer risk, potentially overshadowing or interacting with smoking effects, though larger studies are needed to confirm this.

## Strengths and limitations

The study had several strengths. Controls were selected from cancer patients to ensure comparability and reduce selection bias and participants were blinded to the study hypothesis to minimise recall bias. Key confounders, such as smoking, hot beverage consumption and sociodemographic factors, were controlled by using multivariate logistic regression, enhancing the internal validity of the study.

However, limitations included potential recall bias due to self-reported data, selection bias from recruiting participants at specific cancer clinics and a small sample size due to the limited availability of cancer clinics in Hargeisa, which may affect the generalisability of the results. The small sample size also resulted in wide CIs for some estimates, indicating that this is a preliminary study requiring larger-scale follow-up research to provide more precise risk assessments. Additionally, the case-control design precludes establishing causality, and there may be unmeasured confounders such as dietary factors or alcohol use, although the latter is culturally low in this population. The focus on male participants, necessitated by cultural taboos surrounding female khat chewing disclosure, limits applicability to women.

## Conclusion

The study investigated the relationship between khat chewing and UDT cancers among male patients in Hargeisa, Somaliland. The findings show an association between khat chewing and increased UDT cancer risk, highlighting duration, frequency and amount as key risk factors.

The study urges collaborative efforts among health organisations, government bodies and healthcare providers to develop targeted interventions and policies. These should include public awareness campaigns, cancer registry establishment, cessation programs and regulatory measures like quantity restrictions or taxation. Training for healthcare providers is crucial for early detection and management. Further research on khat's long-term effects and carcinogenic properties is essential to inform evidence-based strategies and protect public health effectively.

## Conflicts of interest

The authors declare no conflicts of interest.

## Funding

This study did not receive any funding.

## Author contributions

AMA: Primary researcher, responsible for conducting the study, data collection, data analysis and writing the article.

MNM: Primary supervisor of the study, overseeing the research process and providing guidance.

AH: Assisted in study design and methodology and consulted on data analysis.

OMM: Assisted with data collection and contributed to the literature review.

## Figures and Tables

**Figure 1.**
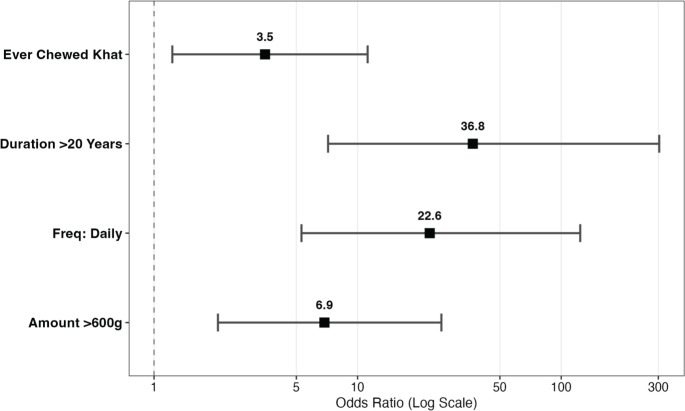
Presents a forest plot summarising the aORs for key khat chewing.

**Table 1.** Association between Khat chewing habits and risk of UDT cancers.

Characteristic	Crude OR (95% CI)	p-value	Adjusted OR (95% CI)	p-value
Khat chewing				
No	Ref		Ref	
Yes	4.84 (1.86–14.4)	0.002	**3.51 (1.23–11.2)**	**0.024**
Duration of khat chewing				
Non-user	Ref		Ref	
<10 years	0.91 (0.17–3.94)	0.90	0.67 (0.11–3.17)	0.63
10–20 years	2.42 (0.68–8.86)	0.17	2.71 (0.68–11.7)	0.16
>20 years	48.3 (10.6–361)	<0.001	**36.8 (7.16–303)**	**<0.001**
Freq of Khat Chewing				
Non-user	Ref		Ref	
≤2 days/week	0.74 (0.10–3.75)	0.74	0.21 (0.01–1.84)	0.23
4 days a week	2.26 (0.64–8.21)	0.20	2.29 (0.61–9.03)	0.22
Every day	25.4 (6.99–116)	<0.001	**22.6 (5.30–124)**	**<0.001**
Amount per session				
Non-user	Ref		Ref	
100–300 g	0.44 (0.02–3.00)	0.47	0.26 (0.01–2.06)	0.26
300–600 g	2.64 (0.69–10.2)	0.15	3.16 (0.74–14.3)	0.12
>600 g	11.1 (3.72–37.9)	<0.001	**6.87 (2.06–25.8)**	**0.002**

CI = confidence interval; OR = odds ratio; Ref = reference group. Crude OR: Unadjusted univariate analysis. Adjusted OR: Multivariate logistic regression adjusted for age, education level, marital status, smoking history and hot beverage consumption. Categories for duration (<5 years and 5–10 years) and frequency (once a week and twice a week) were merged due to small sample sizes to facilitate model convergence.

**Table 2.** Socio-demographic characteristics and behavioural risk factors of cases and controls.

Group	Characteristic	Control (N = 61)	Case (N = 36)	p-value^*^
Socio-demographic characteristics	Age group, *n* (%)			0.65
	18–34 years old	1.0 (1.6%)	0.0 (0.0%)	
	35–54 years old	3.0 (4.9%)	2.0 (5.6%)	
	55–64 years old	20.0 (32.8%)	8.0 (22.2%)	
	>64 years old	37.0 (60.7%)	26.0 (72.2%)	
	Education level, *n* (%)			**0.015**
	No formal education	28.0 (45.9%)	28.0 (77.8%)	
	Primary education	20.0 (32.8%)	5.0 (13.9%)	
	Secondary education	11.0 (18.0%)	2.0 (5.6%)	
	University education	2.0 (3.3%)	1.0 (2.8%)	
	Marital status, *n* (%)			0.40
	Single	11.0 (18.0%)	4.0 (11.1%)	
	Married	46.0 (75.4%)	32.0 (88.9%)	
	Divorced	3.0 (4.9%)	0.0 (0.0%)	
	Widowed	1.0 (1.6%)	0.0 (0.0%)	
	Recruitment site, *n* (%)			0.052
	Needle hospital	31.0 (50.8%)	11.0 (30.6%)	
	Nageeye cancer clinic	30.0 (49.2%)	25.0 (69.4%)	
Khat chewing habits	Ever chewed khat, *n* (%)	31.0 (50.8%)	30.0 (83.3%)	**0.001**
	Duration of chewing, *n* (%)			**<0.001**
	<5 years	3.0 (4.9%)	0.0 (0.0%)	
	5–10 years	13.0 (21.3%)	3.0 (8.3%)	
Khat chewing habits	10–20 years	14.0 (23.0%)	7.0 (19.4%)	
	>20 years	2.0 (3.3%)	20.0 (55.6%)	
	Non-user	29.0 (47.5%)	6.0 (16.7%)	
	Frequency of chewing, *n* (%)			**<0.001**
	Once a week	3.0 (4.9%)	0.0 (0.0%)	
	Twice a week	10.0 (16.4%)	2.0 (5.6%)	
	4 days a week	15.0 (24.6%)	7.0 (19.4%)	
	Every day	4.0 (6.6%)	21.0 (58.3%)	
	Non-user	29.0 (47.5%)	6.0 (16.7%)	
	Amount per session, *n* (%)			**<0.001**
	100–300 g	11.0 (18.0%)	1.0 (2.8%)	
	300–600 g	11.0 (18.0%)	6.0 (16.7%)	
	>600 g	10.0 (16.4%)	23.0 (63.9%)	
	Non-user	29.0 (47.5%)	6.0 (16.7%)	
Other risk behaviours	Current smoker, *n* (%)	26.0 (42.6%)	18.0 (50.0%)	0.48
	Duration of smoking, n (%)			0.084
	<5 years	1.0 (1.6%)	4.0 (11.1%)	
	5–10 years	11.0 (18.0%)	3.0 (8.3%)	
	10–20 years	8.0 (13.1%)	3.0 (8.3%)	
	>20 years	6.0 (9.8%)	8.0 (22.2%)	
	Non-smoker	35.0 (57.4%)	18.0 (50.0%)	
	Frequency of smoking, *n* (%)			0.17
	Once a week	0.0 (0.0%)	0.0 (0.0%)	
	Twice a week	6.0 (9.8%)	1.0 (2.8%)	
	4 days a week	8.0 (13.1%)	3.0 (8.3%)	
	Every day	12.0 (19.7%)	14.0 (38.9%)	
	Non-smoker	35.0 (57.4%)	18.0 (50.0%)	
	Amount (sticks/day), *n* (%)			0.66
	<5 sticks	6.0 (9.8%)	2.0 (5.6%)	
	5–10 sticks	3.0 (4.9%)	3.0 (8.3%)	
	>10	17.0 (27.9%)	13.0 (36.1%)	
	Non-smoker	35.0 (57.4%)	18.0 (50.0%)	
	Consumes hot beverages, *n* (%)	61.0 (100.0%)	35.0 (97.2%)	0.37
	Freq. of hot bev., *n* (%)			0.47
	Once a week	0.0 (0.0%)	1.0 (2.9%)	
	Once every 2–4 days	4.0 (6.6%)	1.0 (2.9%)	
	Once a day	25.0 (41.0%)	12.0 (34.3%)	
	2–3 times a day	32.0 (52.5%)	21.0 (60.0%)	
	Mising	0	1	

*n* = Number of participants; % = Column percentage. *p*-values were calculated using Pearson’s chi-squared test for categorical variables with adequate sample sizes and Fisher’s exact test for variables with small cell counts (expected frequency <5). Percentages may not sum to 100% due to rounding. One participant in the case group had missing data for the frequency of hot beverage consumption. p* <0.05 (Significant)

**Table 3.** Joint effect of Khat chewing and smoking on the risk of UDT cancers.

Characteristic	OR (95% CI)	p-value
Combination of habits		
Neither (no khat, no smoking)	Ref	Ref
Both (chewer & smoker)	**4.69 (1.17–24.4)**	**0.041**
Chewer only	**6.12 (1.51–32.3)**	**0.017**
Smoker only	2.86 (0.41–20.8)	0.28
CI = confidence interval; OR = odds ratio; Ref = reference group. Adjusted OR: multivariate logistic regression adjusted for age group, education level, marital status and hot beverage consumption.		
